# Retinal micro-vascular and aortic macro-vascular changes in postmenopausal women with primary hyperparathyroidism

**DOI:** 10.1038/s41598-018-35017-y

**Published:** 2018-11-08

**Authors:** Jessica Pepe, Cristiana Cipriani, Massimiliano Tedeschi, Mario Curione, Mariacristina Parravano, Monica Varano, Federica Biamonte, Luciano Colangelo, Salvatore Minisola

**Affiliations:** 1 Department of Internal Medicine and Medical Disciplines, “Sapienza” University, Viale del Policlinico 155, 00161 Rome, Italy; 2 IRCCS-G. B. Bietti Foundation, IRCCS, Rome, Italy

## Abstract

Aim of the study was to evaluate the micro and macro-vascular changes in patients with primary hyperparathyroidism (PHPT) compared to controls. 30 postmenopausal PHPT women (15 hypertensive and 15 normotensive) and 30 normotensive controls underwent biochemical evaluation of mineral metabolism and measurements of arterial stiffness by 24 hour ambulatory blood pressure monitoring. Retinal microcirculation was imaged by a Retinal Vessel Analyzer. PHPT patients also underwent bone mineral density measurements and kidney ultrasound. PHPT patients had higher mean calcium and parathyroid hormone values compared to controls. Evaluating macro-vascular compartment, we found higher values of 24 hours-systolic, diastolic blood pressure, aortic pulse wave velocity (aPWV) and aortic augmentation index (Aix) in hypertensive PHPT, but not in normotensive PHPT compared to controls. The eye examination showed narrowing arterial and venular diameters of retinal vessels in both hypertensive and normotensive PHPT compared to controls. In hypertensive PHPT, 24 hours systolic blood pressure was associated only with parathyroid hormone (PTH) levels (beta = 0.36, p = 0.04). aPWV was associated with retinal diameter (beta = −0.69, p = 0.003), but not with PTH. Retinal artery diameter was associated with PTH (beta = −0.6, p = 0.008). In the normotensive PHPT, only PTH was associated with retinal artery diameter (beta = −0.60, p = 0.01) and aortic AIx (beta = 0.65, p = 0.02). In conclusion, we found macro-vascular impairment in PHPT and that micro-vascular impairment is negatively associated with PTH, regardless of hypertension in PHPT.

## Introduction

In primary hyperparathyroidism (PHPT), a combination of structural and functional vascular changes has been advocated with conflicting results^1^. Thus, currently, the presence of atherosclerosis, even with early vascular abnormalities or so called vascular impairments, is not included among the criteria needed to submit patients to surgery^2^. Few studies reported that these vascular impairments in PHPT were associated with hypertension^3,4^, while others reported that they were related to high calcium and parathyroid hormone levels which are the biochemical features of PHPT^5–7^. Other authors did not find any difference between PHPT and controls as regards vascular functions^8,9^.

These results may be due to different calcium levels in the populations studied, or to co-morbidities or also to different methods used to measure vascular impairment, namely arterial stiffness.

Currently, regional and local arterial stiffness may be measured directly and noninvasively, at various sites along the arterial tree, by assessing pulse wave velocity (PWV) and augmentation index (AIx)^10^. Aortic PWV has been shown to be a powerful predictor of cardiovascular events in the general population^11^. We chose 24 hour ambulatory blood pressure monitoring (ABPM) to determine both PWV and Aix, because this method has never been utilized in PHPT populations^12^. Interestingly, studies suggest that large artery stiffness is also associated with micro-vascular changes^13^. In particular, over the last decade, a large population–based study has shown that very small vessels, such as in the retina, are associated with a wide range of subclinical and clinical cardiovascular disease^13,14^. The retina represents a unique site where the microvasculature can be directly and non-invasively visualized, but until now, with a limit of subjective clinical assessment. However, nowadays a modern digital imaging systems have revolutionized the assessment of retinal photograph by capturing a larger segment of the retina and allowing a more objective documentation. So far, there is no information available regarding retinal micro-vascular assessment in PHPT patients.

Our study aimed to provide a preliminary analysis of the micro-vascular retinal vessels using a modern digital imaging system and of the macro-vascular compartment using 24 hour ABPM to derive both PWV and Aix in PHPT patients with and without arterial hypertension compared to controls.

## Results

The characteristics of PHPT patients are reported in Table 1. Seventy - three percent of subjects had densitometric osteoporosis; morphometric or clinical vertebral fracture were present in 16% of the patients and 13% of the patients reported peripheral fractures. Ultrasound revealed renal calculi in 23% of the patients. Serum calcium levels higher than 1 mg/dl above the normal range, were present in 13% of the patients. All patients, met the current criteria for surgery according to current guidelines, except three patients with mild disease^2^.

**Table 1:** Anthropometric and biochemical parameters in patients with primary hyperparathyroidism (PHPT) hypertensive and normotensive and controls.

	Controls (n = 30)	PHPT Hypertensive (n = 15)	PHPT Normotensive (n = 15)
Age (years)	61.0 ± 9.8	61.1 ± 4.8	64.8 ± 7.8
Years since menopause (years)	12.4 ± 8.9	15.2 ± 6.2	14.7 ± 7.18
Body mass index (kg/m^2^)	23.1 ± 2.8	24.9 ± 3.6	23.9 ± 4.2
Creatinine (mg/dl)	0.8 ± 0.1	0.8 ± 0.2	0.7 ± 0.1
Calcium (mg/dL)	9.3 ± 0.4	10.7 ± 0.5**	10.6 ± 0.3^§§^
Ionized Calcium (mmol/L)	1.2 ± 0.0	1.4 ± 0.1***	1.4 ± 0.0^§§^
Phosphorus (mg/dL)	4.0 ± 0.3	2.9 ± 0.3**	2.9 ± 0.4^§§^
25(OH)D (nmol/L)	34.6 ± 11.4	37.1 ± 11.3	36.4 ± 14.3
Urinary calcium 24 hours	214.0 ± 62.2	264.3 ± 84.7*	274.1 ± 122.0^§^
PTH (ng/L)	36.4 ± 14.3	100.4 ± 37.4**	80.1 ± 26.3^§§^
Alkaline phosphatase (U/L)	82.2 ± 23.3	91.5 ± 47.0	83.4 ± 54.7
Glucose (mg/dL)	89.8 ± 6.0	86.7 ± 5.6	88.7 ± 6.5
Total cholesterol (mg/dL)	203.8 ± 31.4	210.6 ± 40.8	190.9 ± 30.0
High Density Lipoprotein (mg/dL)	61.1 ± 13.2	62.2 ± 10.6	57.1 ± 10.3
Low Density Lipoprotein (mg/dL)	121.1 ± 27.5	128.5 ± 25.8	114.9 ± 22.6
Triglycerides (mg/dL)	120.6 ± 38.1	115.13 ± 42.08	124.4 ± 60.7

Results are presented as mean ± 1 SD. Controls vs PHPT hypertensive *p ≤ 0.05; **p ≤ 0.0001, ***p ≤ 0.00001. Controls vs PHPT normotensive ^§^p ≤ 0.05; ^§§^p ≤ 0.0001.

There were no difference between PHPT and controls considering anthropometric parameters, nor between hypertensive PHPT patients and normotensive PHPT patients (Table 1).

As expected, mean total and ionized calcium, parathyroid hormone (PTH) and 24 hour urinary calcium were higher in both groups of PHPT patients compared to controls (all p < 0.001, Table 1).

No differences were found as regards biochemical evaluation between hypertensive and normotensive PHPT patients.

Cardiovascular risk factors were similar between PHPT patients and controls regarding BMI, glucose and lipid profile as reported in Table 1. Alcohol consumption was ascertained in 13.3% of the PHPT population and in 16.6% of the control subjects (p = ns). Tobacco use was recorded in 20% of the PHPT population and in 26.6% of the control subjects (p = ns).

Twelve PHPT patients affected by hypertension were under medication. In particular, 9 patients were under alfa-blockers and 3 were under loop-diuretics. The mean time since diagnosis of hypertension was 5 ± 2 years.

We found that hypertensive PHPT patients had higher values of 24 h-systolic, diastolic blood pressure and aortic systolic blood pressure (SBP-Ao) as compared to the controls and to the normotensive PHPT group (all p < 0.05, Table 2). Seven out of fifteen PHPT patients showed a non physiological decrease in blood pressure during night time (non dipper).

**Table 2:** 24 hour parameters of blood pressure, macro-vascular parameters of arterial stiffness and retinal micro-vascular parameters in hypertensive and normotensive patients with primary hyperparathyroidism (PHPT) and controls.

	Controls (n = 30)	PHPT Hypertensive (n = 15)	PHPT Normotensive (n = 15)
**24 hour ambulatory blood pressure**			
24 hour -SBP (mmHg)	116.03 ± 13.71	153.26 ± 6.16**	123.46 ± 7.04^°°°^
24 hour –DBP (mmHg)	73.30 ± 14.54	88.26 ± 10.93**	70.2 ± 8.3^°°^
MAP (mmHg)	87.37 ± 9.14	109.06 ± 7.14**	88.6 ± 8.79^°°^
ABPM-PP (mmHg)	51.18 ± 20.77	71.86 ± 10.02*	51.80 ± 9.06^°°^
SBP-AO (mmHg)	118.12 ± 15.48	156.33 ± 8.10**	120.8 ± 10.31^°°^
HR (beats/minutes)	73.30 ± 14.49	71.0 ± 6.69	72.8 ± 8.3
**Macro-vascular assessment**			
Brachial AIx (%)	−22.15 ± 22.43	−17.62 ± 8.80	−22.71 ± 11.43
Aortic AIx (%)	28.74 ± 9.87	42.62 ± 10.88*	30.01 ± 13.67^°^
aPWV (m/s)	7.53 ± 2.70	10.81 ± 1.00**	7.97 ± 1.26^°°^
**Micro-vascular assessment**			
Retinal artery diameter (μm)	109.47 ± 11.91	99.33 ± 16.53*	101.00 ± 15.37^§^
Retinal vein diameter (μm)	133.57 ± 14.40	124.19 ± 19.99*	125.6 ± 14.47^§^
AVR (index)	0.86 ± 0.05	0.83 ± 0.06	0.82 ± 0.06^§^

Results are presented as mean ± 1 SD, or percenteges.

Legend: SBP = systolic blood pressure, DBP = diastolic blood pressure, MAP = mean arterial pressure, PP = pulse pressure, AO = aortic, HR = heart rate, Aix = arterial index, aPWV = aortic pulse wave velocity, AVR = retinal artery vein ratio.

*p ≤ 0.05; **p ≤ 0.0001 Controls vs PHPT hypertensive.

^§^p ≤ 0.05; ^§§^p ≤ 0.0001 Controls vs PHPT normotensive.

^°^p ≤ 0.05, ^°°^p ≤ 0.0001, ^°°°^p ≤ 0.0001 PHPT hypertensive vs PHPT normotensive.

Mean baseline best corrected visual acuity was 0.1 ± 0.1 LogMar with a mean refractive error of +1.25 sf.

In the assessment of the macro-vascular compartment, aortic PWV and aortic AIx were significantly higher in hypertensive PHPT patients as compared to both the controls and to the normotensive PHPT patients (all p < 0.05, Table 2).

The normotensive PHPT patients showed no difference as concerns blood pressure and macrovascular parameters compared to the controls.

The eye examination to explore the micro-vascular compartment showed a narrowing arterial and venular diameter of the retinal vessels in both hypertensive and normotensive PHPT patients compared to the controls (Table 2).

**Figure 1:**
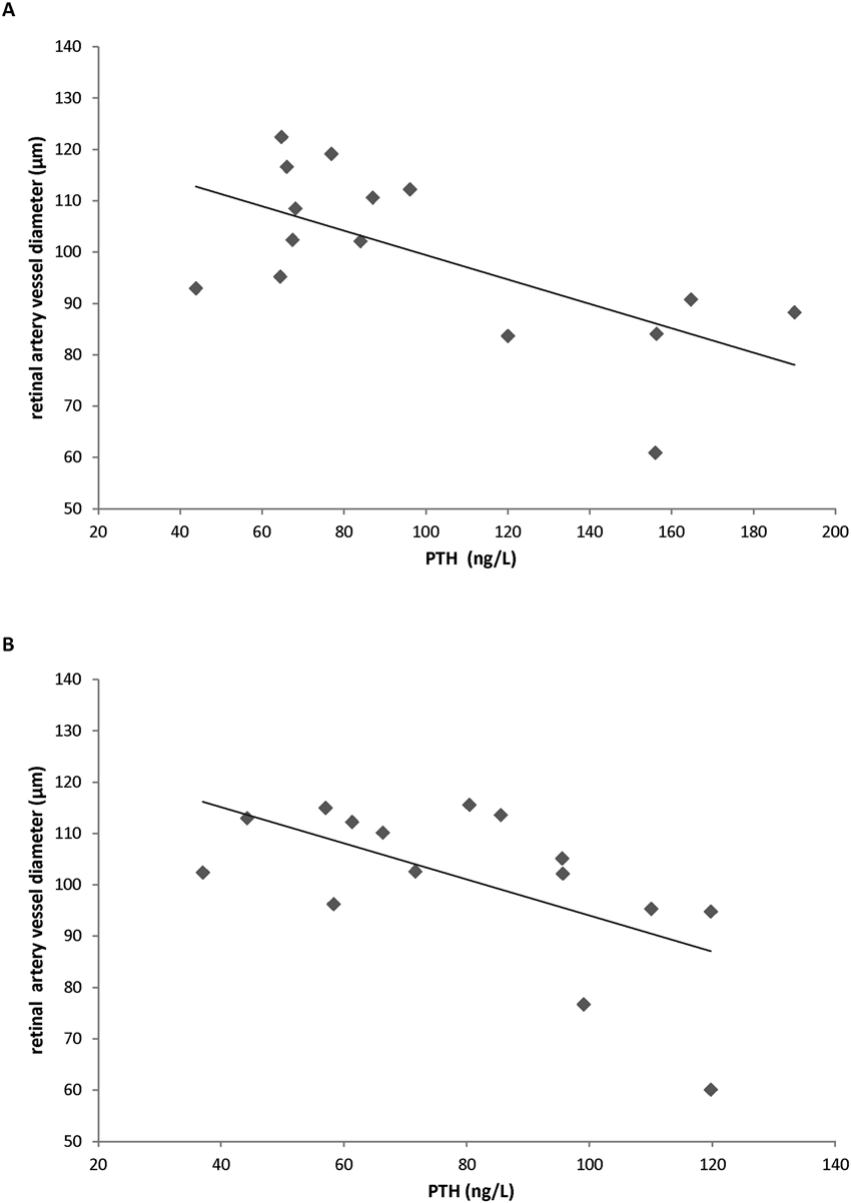
Association between retinal artery vessel diameter and PTH levels in hypertensive PHPT patients (**A**) and normotensive PHPT patients (**B**).

We found that 24 h systolic blood pressure and SBP-Ao were associated with PTH levels in the PHPT hypertensive group (beta = 0.36, p = 0.04 and beta = 0.71, p = 0.002 respectively), but not with age, BMI and calcium levels. aPWV was associated with retinal arterial diameter (beta = −0.69, p = 0.003), but not with PTH, BMI, blood pressure and age. Retinal arterial diameter was negatively associated with PTH (beta = −0.60, p = 0.008, Fig. 1A).

**Figure 2:**
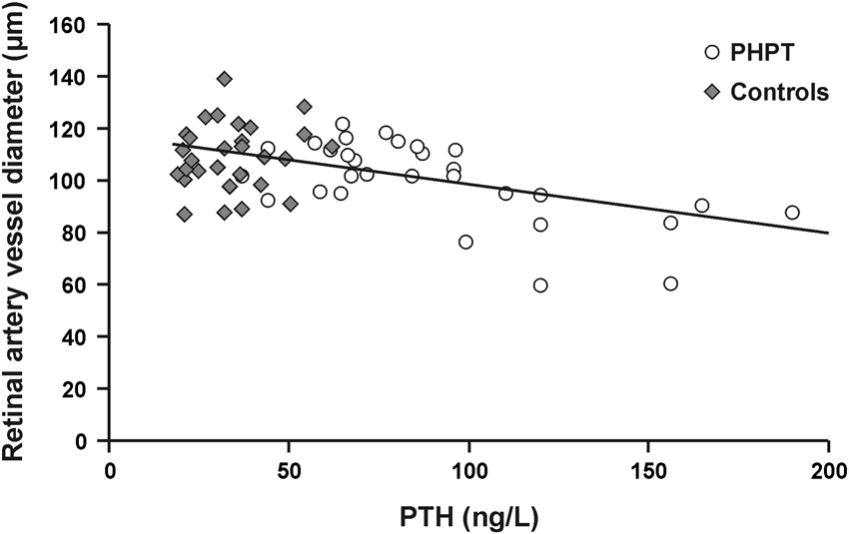
Association between retinal artery vessel diameter and PTH levels in all the sample studied including PHPT patients and controls.

In the normotensive PHPT group, we did not find an association between aPWV and retinal vessel diameter, nor with PTH neither with blood pressure parameters. The only associations found were a negative association between PTH and retinal arterial diameter (beta = −0.60, p = 0.01, Fig. 1B), and a positive association between PTH and aortic AIx (beta = 0.65, p = 0.02). Considering all of the PHPT sample and including controls, a negative association between retinal arterial vessel diameter and PTH was also found (beta = −0.51, p < 0.001, Fig. 2).

## Discussion

The strength of this study derives from the demonstration for the first time of a retinal vessel impairment in PHPT patients together with macro-vascular abnormalities in hypertensive PHPT patients and a retinal vessel narrowing in normotensive PHPT patients, compared to controls. In particular, in PHPT patients with and without hypertension PTH levels are negatively associated with retinal vessel diameter.

The macro-vascular abnormalities shown in our study are in line with previous papers on this topic^3,4^, while the novelty of micro-vascular retinal vessel narrowing in PHPT, regardless of the presence of hypertension, deserves attention. Indeed, in the general population, retinal arteriolar narrowing may precede the presentation of hypertension by years in initially normotensive individuals, supporting the view that micro-vascular changes exert a key role in the evolution from normal to elevated blood pressure^15^. The atherosclerosis risk in communities study showed that normotensive participants, who had generalized arteriolar narrowing at baseline, were 60% more likely to be diagnosed with hypertension over a 3-year period than individuals who did not have arteriolar narrowing^16^.

The pathophysiological mechanisms that link PTH to vascular compliance markers remain unclear. PTH may influence arterial vasculature through the serum levels modification of several molecules such as osteoprotegerin or vessel endothelial growth factor^17–21^. Another plausible explanation is that PTH adversely affects vascular smooth muscle cells, which might increase vascular stiffness and promote atherosclerotic changes^22,23^. Evidence from animal and human studies suggests that endothelium is an important regulator of arterial stiffness and discussions are still ongoing as to whether endothelial dysfunction contributes to increased arterial stiffness or if arterial stiffness causes endothelial alteration, which in turn further increases arterial stiffness.

Considering macro-vascular damage, we observed a higher aPWV in hypertensive PHPT patients, but not a higher brachial AIx compared to controls and normotensive PHPT patients.

The AIx is the proportion of central pulse pressure that results from arterial wave reflection and is a commonly used measure of arterial stiffness. Although the timing of the arrival of the reflected wave at the proximal aorta is largely determined by large artery PW, Aix is not a simple surrogate measure of PWV. It is influenced by vasoactive drugs independently of aPWV^24^ suggesting that it is also determined by the intensity of wave reflection which, in turn, is determined by the diameter and elasticity of small arteries and arterioles. However, twin studies suggest that AIx is partially heritable and independent of these variable^25^. It might be possible that in our sample the use of antihypertensive drugs has influenced this parameter; however studying hypertensive patients without any medication would have been unethical.

It should be noted that at the moment in Europe and in America there is no specific practical recommendation on the use of PWV and AIx and more data are required before these vascular biomarkers can be recommended for routine use in the general population^12^.

However, PWV is a well know factor of increased cardiovascular risk in the general population and it might be possible that increased vascular stiffness in subjects with PHPT may account in part for the increased cardiovascular risk in this group.

Our study has a few limits. We studied only postmenopausal women of a single geographical area. Thus, men and PHPT patients in different countries may show other clinical expressions, as it has been shown for the classical manifestations of the disease^26,27^.

Taken together our data point to the role of PTH as a marker of atherosclerosis in PHPT patients not only for large vessels but also for small vessels, as it has been suggested also in the general population^28^.

Moreover our study suggests that the eye examination, which is a non invasive and well know tool for the examination of hypertensive patients, may have a possible role also in the clinical assessment of normotensive PHPT, as retinal vessels are a possible target of PTH. We considered in the current study the assessment of a single point of the retinal temporal artery and of the retinal temporal vein as representative of the changes in the retinal circulation. However more generalized indexes, such as the central retinal arterial equivalent and venular equivalent, might be more suitable indicators of peripheral micro-vascular abnormalities.

Further longitudinal studies are needed to determine the temporal relationship between the development of diminished vascular plasticity and possible incident cardiovascular events in PHPT patients. Moreover, since we studied patients who met surgical criteria for parathyroid adenoma removal, interest arises, for further research, as to whether surgical treatment may influence both macro and micro-vascular abnormalities in PHPT patients.

## Methods

### Subjects

The population studied included 15 consecutive Caucasian postmenopausal women (age range 55–75 years old) diagnosed as having PHPT with hypertension and 15 without hypertension at the Metabolic Bone Disease Unit of the University of Rome, ‘Sapienza’ (Italy), between January 2017 and February 2018. The diagnosis of PHPT was made following the finding of hypercalcemia with an inappropriately high plasma level of PTH^29^. During the same period, in the same center, 30 Caucasian volunteer postmenopausal women in the same age range and with the same body mass index (BMI), without hypertension, were enrolled as the control group. Exclusion criteria included: BMI > 30 kg/m^2^, hypo- and hyperthyroidism, previous cardiovascular diseases, previous medical history of dyslipidemia and/or use of statins, use of beta blocker, angiotensin-converting-enzyme inhibitor, estrogen replacement therapy, diabetes mellitus type 1 and 2, creatinine clearance <60 ml/min and  history of peripheral arterial diseases.

We excluded eyes diseases which could modify retinal vessels, especially a history of glaucoma, retinal vein occlusion and retinitis pigmentosa. In addition patients with spherical refractive error ≥±6 diopters, astigmatism ≥±3 diopters, optical media opacities that could influence functional and structural retinal testing were excluded.

Every patient provided written informed consent. The study was approved by “Policlinico Umberto I” Hospital Ethics Committee (protocol number 3454) and by the Institutional Review Board of the G.B. Bietti Foundation. The study protocol adhered to the tenets of the Declaration of Helsinki.

After informed consent was obtained, patients and control subjects with 25(OH)D levels lower than 20 ng/mL were treated weekly with 50,000 I.U. of cholecalciferol for one month. After completing a validated questionnaire, those on a calcium poor diet were supplemented with calcium (1,000 mg) per os daily, which is the recommended dose^30^. After one month of supplementation, both PHPT and controls were reevaluated by blood sample analysis.

### Cardiovascular risk factors

BMI was calculated as weight (kilograms) divided by the square of height (square meters). Alcohol consumption (more than three alcoholic units per day) and current smoking status were assessed in a face-to-face medical interview. Blood pressure (millimetres of mercury) was measured after 5 minutes of rest, at the upper left arm with subjects in a lying position and using an oscillometric blood pressure (BP) monitor to diagnose hypertension, following the current European Society of Cardiology guidelines^31^.

### Biochemical evaluation

We measured total and ionized calcium, phosphorus, creatinine, glucose, 25(OH) vitamin D, parathyroid hormone (PTH), alkaline phosphatase, as described previously^32,33^. Creatinine clearance (CrCl) was calculated using the formula of Cockcroft and Gault. Total cholesterol, high density lipoprotein (HDL) and triglycerides (TC) were measured using enzymatic methods (Hitachi 911 analyzer, Roche Diagnostics, Basel, Switzerland). LDL was calculated with the Friedewald Formula: LDL = TC − HDL − TG/5.0 (mg/dL).

### Skeletal and renal assessment

In PHPT patients, both skeletal and renal assessment were carried out to better characterize the disease. Bone mineral density (BMD) of the lumbar spine (L1–L4) in the anterior–posterior projection, of the femur (neck and total hip) and nondominant distal one-third radius was measured in each PHPT patient by dual-energy X-ray absorptiometry (Hologic QDR 4500, Hologic Inc, Waltham, MA, USA). The precision error of lumbar spine and total hip measurements was 1.3 and 1.7%, respectively and 1.3% at the distal 1/3 radius^34^. Fractured lumbar vertebrae were excluded from BMD measurement. Each patient subject underwent morphometric vertebral assessment (VFA) with the DXA scan^35^. Abdominal ultrasound was performed by a skilled radiologist to detect renal calculi, with an ultrasound scanner (Esaote MyLab 70 x Vision; Genoa, Italy), as previously described^36^.

### Non-invasive vascular studies

All participants underwent 24 hour ABPM. They were instructed to keep their non-dominant arm still at the side during measurements. Measurements were carried out by using the 24 hour TensioMed Arteriograph system (TensioMed Ldt., Budapest, Hungary). The automatic monitoring system was set up to obtain measurements every 15 minutes while subjects were awake and every 30 minutes while the subjects were sleeping. Details of this device have been previously described^12^. This technique is based on the fact that the contraction of the myocardium generates pulse waves in the aorta. The pulse wave goes to the arm (first wave), where the cuff is located, then to the aorta. Part of the wave is reflected at the bifurcation of the aorta, goes back, adds to the first and is sensed by the cuff. The first wave is reflected at the bifurcation of the aortic wall; a second, reflected wave therefore appears as a late systolic peak.

The morphology of this second wave depends on the stiffness of the large artery, the reflection time at 35 mmHg suprasystolic pressure of the brachial artery and the peripheral resistance-dependent amplitude. The cuff collects a pulse wave that contains both the first and reflected waves. The software calculates aortic PWV (aPWV) and AIx from this combined wave. The speed with which the pulse wave travels along the jugular fossa-symphysis distance is aPWV. The jugular fossa–symphysis distance is anatomically identical to the distance between the aortic trunk and the bifurcation. Because PWV indicates the pulse wave speed in the arterial tree: if the artery is stiff, the speed is increased. The AIx is defined as the percentage ratio of the pressure increment caused by the reflected wave to the direct wave. Normally, the reflected component in peripheral waves is always smaller than the direct component and AIx is negative.

### Retinal vessel assessment

In order to exclude other diseases that may modify retinal vessel diameter, each patient received a comprehensive ophthalmological assessment which involved a visual acuity and a slit-lamp examination, a Goldmann applanation tonometry and an ocular fundus evaluation.

All patients and controls were imaged by a Retinal Vessel Analyzer (RVA) by positioning the subject in front of a non-midriatic camera (TopconTRC-NV2000) to obtain a retinographic image (45°). The images were then processed by IMEDOS software measuring the caliber of vessels emerging from the optic disc, as previously described^37^. In particular, the RVA produces continuous and automated analysis of the vessels combined with correction for small eye movements. Using an adaptive algorithm to calculate the size of the vessels (Vessel Map 2-Z), this system combines high reproducibility with high temporal and of spatial resolution.

**Figure 3:**
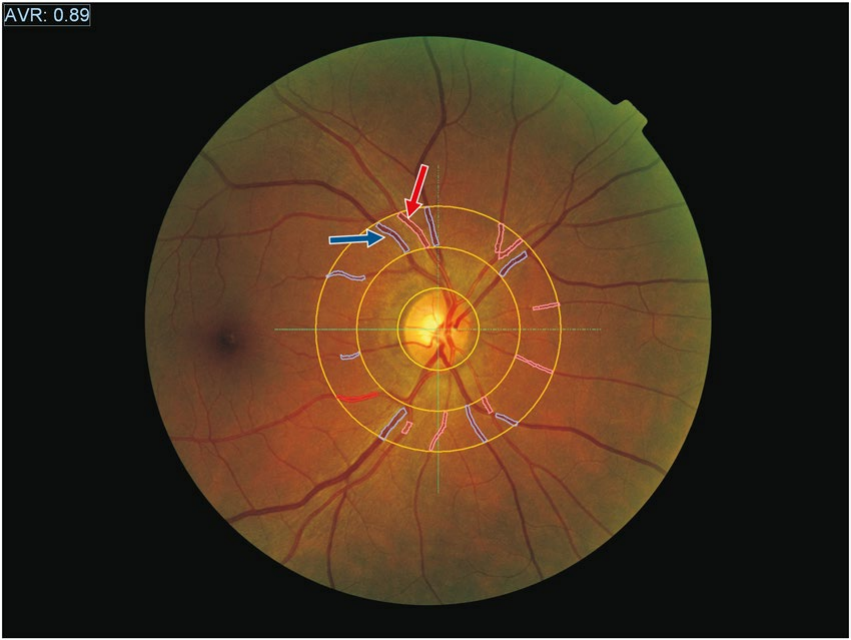
Image of an eye examination in a PHPT patient, obtained by retinal vessel analyzer. The assessments of the arterial caliber and of the vein caliber were taken between 1 and 2 disc diameters from the margin of the optic disc, in correspondence with the major superior temporal arteries (red arrow) and vein (blue arrow) of the right eye.

Measurements of the right eye were taken: in particular superior temporal retinal artery diameter (RAD), superior temporal retinal vein diameter (RVD) and artery-vein ratio (AVR) were taken between 1 and 2 disc diameters from the margin of the optic disc major (Fig. 3). Generally we considered the more temporal vein as “superior temporal vein” and the other vessel was recognized as a medial retinal vein or tributary vessel of the superior temporal vein^38^.

In view of the high reproducibility of the procedure, only one acquisition session was scheduled for each subject. One single measurement was performed by positioning the caliber at the outer margin of the first circle delimitating the optic disc^39^, due to the results of a previous study demonstrating the high correlation in vascular morphology between the left and the right eye^40^.

### Statistical analysis

Statistical analysis was performed using SPSS for Windows version 13 (SPSS Inc., Chi, IL, USA). Categorical variables are described with numbers and percentages. Continuous variables are expressed as mean value ± SD. Differences between groups were assessed with chi-squared test for categorical variables. We checked all variables in the three groups for normality. For comparison of continuous variables that failed to pass normality test, we used the Mann – Whitney test. For comparison of continuous variables was made using unpaired t-test. Pearson correlation coefficient (beta) was calculated for the measurement of micro-vascular and macro-vascular parameters with biochemical and anthropometric values. A p value < 0.05 was considered statistically significant. As there are no data on retinal vessels in PHPT, it was not possible to calculate a sample size; thus, we decided to include at least 15 hypertensive patients, considering the previous study where 15 patients affected by PHPT and hypertension had a significantly different AIx compared to controls^6^.
